# A phase 1 study of filanesib, carfilzomib, and dexamethasone in patients with relapsed and/or refractory multiple myeloma

**DOI:** 10.1038/s41408-019-0240-6

**Published:** 2019-10-01

**Authors:** Hans C. Lee, Jatin J. Shah, Lei Feng, Elisabet E. Manasanch, Rebecca Lu, Ashley Morphey, Brandon Crumpton, Krina K. Patel, Michael L. Wang, Raymond Alexanian, Sheeba K. Thomas, Donna M. Weber, Robert Z. Orlowski

**Affiliations:** 1 Department of Lymphoma/Myeloma, The University of Texas MD Anderson Cancer Center, Houston, TX USA; 2 Karyopharm Therapeutics, Newton, MA USA; 3 Department of Biostatistics, The University of Texas MD Anderson Cancer Center, Houston, TX USA; 4 Department of Experimental Therapeutics, The University of Texas MD Anderson Cancer Center, Houston, TX USA

**Keywords:** Myeloma, Myeloma

The use of novel agents including immunodulatory drugs (IMiDs), proteasome inhibitors (PIs), and more recently anti-CD38 monoclonal antibodies has produced a substantial improvement in response rates and response durability in multiple myeloma patients. However, myeloma remains largely incurable, and the majority of patients eventually become resistant to all available therapies, which highlights the need to develop new drugs and rational combinations with distinct mechanisms of action.

Kinesin spindle protein (KSP, *KIF11*) is a mitotic motor kinesin that plays an essential role in mediating centrosome separation and maintenance of spindle bipolarity during the early stages of mitosis^1^. In particular, hematologic malignancies such as multiple myeloma may be particularly susceptible to KSP inhibition due to their dependence on short-lived anti-apoptotic proteins such as myeloid cell leukemia 1 (Mcl-1) which are not transcribed or translated during mitotic arrest yet still undergo proteolysis^2,3^.

In a phase 1 trial in relapsed and/or refractory multiple myeloma, the safety and preliminary efficacy of filanesib (ARRY-520), a potent, selective KSP inhibitor, was demonstrated both as a single-agent and in combination with dexamethasone, where overall response rates were 15% and 16%, respectively, at the maximum tolerated dose (MTD) of 1.5 mg/m^2^ on days 1 and 2 administered every 14 days^4^. The most common dose-limiting toxicities (DLTs) were febrile neutropenia and mucositis, and notably, prophylactic filgrastim was mandated for the trial. The combination of filanesib, bortezomib, and dexamethasone was also evaluated in a phase 1 study which demonstrated a 29% response rate in the subset of patients refractory to PIs such as bortezomib, suggesting that there may be synergy between PIs and filanesib. Based on this rationale, we conducted a two-part phase 1 study of filanesib in combination with the second generation PI carfilzomib and dexamethasone in relapsed and/or refractory multiple myeloma with ≥1 prior line of therapy (NCT01372540).

Each part of the study (Part A and Part B) consisted of a separate 3 + 3 dose-escalation of filanesib and carfilzomib, followed by a dose-expansion at the maximum tolerated dose (MTD) of each drug (see Supplementary Methods, Supplementary Fig. S1, and Supplementary Table S1). In Part A, filanesib was administered intravenously (IV) on days 1, 2, 15, and 16 in escalating doses starting at 0.75 mg/m^2^ with fixed-dose carfilzomib 20 mg/m^2^ IV on days 1 and 2 of cycle 1 only, and 27 mg/m^2^ on days 8, 9, 15, and 16, and for all subsequent days of each cycle thereafter. Dexamethasone 4 mg was administered prior to each carfilzomib infusion. In Part B of the study, filanesib was administered at a fixed dose on days 1, 2, 15, and 16 at the MTD determined from Part A in combination with escalating doses of carfilzomib, beginning at 20 mg/m^2^ on days 1 and 2 of cycle 1 only, and 36 mg/m^2^ on days 8, 9, 15, and 16, and 36 mg/m^2^ for all subsequent days of each cycle thereafter. For the Part B dose-expansion, the protocol was amended mid-study to increase dexamethasone to 40 mg on days 1, 8, and 15, although this impacted only four patients on study who increased their dexamethasone dose after cycle 1 (*N* = 1) or cycle 3 (*N* = 3).

After eight cycles of therapy, patients transitioned to a maintenance carfilzomib dosing schedule on days 1, 2, 15, and 16, while continuing filanesib and dexamethasone dosing as per the prior treatment cycles. Prophylactic use of granulocyte-colony stimulating factor (G-CSF) with filgrastim was mandated for 3–7 days starting on day 3 or 4 and on day 17 or 18 of each cycle.

Safety and myeloma disease evaluations were performed prior to each cycle, and disease response and progression were assessed as per International Myeloma Working Group (IMWG) Uniform Response Criteria. The Kaplan–Meier method was used to estimate time-to-event outcomes including progression-free survival (PFS) and overall survival (OS) (see Supplementary Methods).

Between March 1, 2012 and May 31, 2016, a total of 64 relapsed and/or refractory multiple myeloma patients with a median of five lines of prior therapy were enrolled and treated on study. Baseline patient characteristics are summarized in Supplementary Table S2. A total of 48 patients (75%) were refractory to lenalidomide, 37 patients (58%) were refractory to bortezomib, 22 patients (34%) were refractory to carfilzomib, and 40 patients (63%) were double refractory to IMiDs (lenalidomide and/or pomalidomide) and proteasome inhibitors (bortezomib and/or carfilzomib). In patients with available cytogenetics/fluorescence in situ hybridization (FISH) data, high-risk cytogenetics defined as deletion 17p, t(4;14) and/or t(14;16) as per Revised International Staging System (R-ISS) criteria^5^ were present in 6 of 48 patients (13%) and in 17 out of 49 patients (35%) when expanding the definition of high-risk disease to include patients with +1q21, −1p, hypodiploidy, and/or deletion 13q by conventional cytogenetics^6^.

During the Part A 3 + 3 dose-escalation of filanesib in combination with fixed-dose carfilzomib and dexamethasone, filanesib 1.5 mg/m^2^, carfilzomib 20/27 mg/m^2^, and dexamethasone 4 mg was determined to be the MTD (1 DLT among 6 DLT-evaluable patients) and chosen for the dose-expansion cohorts of Part A of the study. During the Part B 3 + 3 dose-escalation of carfilzomib with fixed dosed filanesib 1.5 mg/m^2^ and dexamethasone, the MTD was determined to be filanesib 1.5 mg/m^2^, carfilzomib 20/56 mg/m^2^, and dexamethasone 40 mg weekly (0 DLTs among 6 DLT-evaluable patients) and chosen for the Part B dose-expansion cohort.

Among 30 patients treated at the MTD for the Part A dose-expansion cohorts with filanesib 1.5 mg/m^2^, carfilzomib 20/27 mg/m^2^, and dexamethasone 4 mg, the median number of cycles of treatment was 7 (range 1–44), and median time on therapy was 6.2 months. Most common grade 3 and 4 hematologic AEs were neutropenia (37%/43%), thrombocytopenia (43%/17%), leukopenia (43%/17%), and anemia (33%/3%) (Supplementary Table S3). Most frequent grade 3 and 4 non-hematologic AEs included elevated lipase (13%/0%), dyspnea (10%/0%), fatigue (10%/0%), and pneumonia (10%/0%). In all four cases of grade 3 lipase elevation, the patients were asymptomatic without clinical symptoms of pancreatitis.

A total of 14 patients were treated at the MTD for the Part B dose-expansion cohort with filanesib 1.5 mg/m^2^, carfilzomib 20/56 mg/m^2^, and dexamethasone (4 mg prior to each carfilzomib dose in 10 patients and 40 mg on days 1, 8, and 15 in 4 patients due to protocol amendment). Patients received a median of four cycles (range 1–11) of treatment, and the median duration of therapy was 4.2 months. The most frequent treatment-emergent hematologic grade 3 and 4 AEs were thrombocytopenia (50%/22%), leukopenia (22%/17%), neutropenia (11%/22%), and anemia (22%/0%). The most frequent grade 3 and 4 non-hematologic AEs included dyspnea (11%/0%). Among all patients treated on study, there was one death while on study in a patient treated in the Part A dose-escalation at dose level 3 which was attributed to neutropenic fever and presumed infection.

Among 63 response-evaluable patients in the study, the overall response rate (ORR, ≥partial response) was 37% including 29% partial response (PR) and 7% very good partial response (VGPR) (Table 1a). In patients who were carfilzomib-refractory, dual-refractory to a PI and IMiD, or had ≥3 prior lines of therapy, the ORR was 14%, 26%, and 31%, respectively. Response to therapy was comparable in patients treated at the Part A MTD dose levels as to the overall study population (Table 1b). Among 14 patients treated at the Part B MTD dose levels (Table 1c), ORR was 50% and CBR was 79%. In patients who were carfilzomib-refractory, dual-refractory to a PI and IMiD, or had ≥3 prior lines of therapy, the ORR was 40%, 50%, and 56%, respectively, although results should be interpreted with caution due to the small sample size in these subsets.

**Table 1:** Overall response in (a) all patients, (b) patients treated at Part A MTD, and (c) patients treated at Part B MTD

	All *N* = 63	Cfz-Ref *N* = 21	PI/IMiD-Ref *N* = 39	1–2 lines *N* = 12	≥3 lines *N* = 51	High-risk^a^ *N* = 17
(a) Overall response (all patients)						
sCR/CR, *N* (%)	0 (0%)	0 (0%)	0 (0%)	0 (0%)	0 (0%)	0 (0%)
VGPR, *N* (%)	5 (8%)	1 (5%)	3 (8%)	1 (8%)	4 (8%)	0 (0%)
PR, *N* (%)	18 (29%)	2 (10%)	7 (18%)	6 (50%)	12 (24%)	2 (12%)
MR, *N* (%)	8 (13%)	3 (14%)	5 (13%)	3 (25%)	5 (10%)	5 (29%)
ORR (≥PR), *N* (%)	23 (37%)	3 (14%)	10 (26%)	7 (58%)	16 (31%)	2 (12%)
CBR (≥MR), *N* (%)	31 (49%)	6 (29%)	15 (38%)	10 (83%)	21 (41%)	7 (41%)

	All *N* = 29	Cfz-Ref *N* = 9	PI/IMiD-Ref *N* = 20	1–2 lines *N* = 4	≥3 lines *N* = 25	High-risk^a^ *N* = 7
(b) Overall response (Part A MTD)						
sCR/CR, *N* (%)	0 (0%)	0 (0%)	0 (0%)	0 (0%)	0 (0%)	0 (0%)
VGPR, *N* (%)	3 (10%)	0 (0%)	2 (10%)	0 (0%)	3 (12%)	0 (0%)
PR, *N* (%)	7 (24%)	1 (11%)	2 (10%)	3 (75%)	4 (16%)	1 (14%)
MR, *N* (%)	1 (3%)	0 (0%)	0 (0%)	0 (0%)	1 (4%)	1 (14%)
ORR (≥PR), *N* (%)	10 (34%)	1 (11%)	4 (20%)	3 (75%)	7 (28%)	1 (14%)
CBR (≥MR), *N* (%)	11 (38%)	1 (11%)	4 (20%)	3 (75%)	8 (32%)	2 (29%)

	All *N* = 14	Cfz-Ref *N* = 5	PI/IMiD-Ref *N* = 8	1–2 lines *N* = 5	≥3 lines *N* = 9	High-risk^a^ *N* = 6
(c) Overall response (Part B MTD)						
sCR/CR, *N* (%)	0 (0%)	0 (0%)	0 (0%)	0 (0%)	0 (0%)	0 (0%)
VGPR, *N* (%)	1 (7%)	1 (20%)	1 (13%)	0 (0%)	1 (11%)	0 (0%)
PR, *N* (%)	6 (43%)	1 (20%)	3 (38%)	2 (40%)	4 (44%)	0 (0%)
MR, *N* (%)	4 (29%)	2 (40%)	3 (38%)	2 (40%)	2 (22%)	4 (67%)
ORR (≥PR), *N* (%)	7 (50%)	2 (40%)	4 (50%)	2 (40%)	5 (56%)	0 (0%)
CBR (≥MR), *N* (%)	11 (79%)	4 (80%)	7 (88%)	4 (80%)	7 (78%)	4 (67%)

*Cfz* carfilzomib, *PI* proteasome inhibitor, *IMiD* immunodulatory drug, *sCR* stringent CR, *CR* complete response, *VGPR* very good partial response, *PR* partial response, *MR* minimal response, *CBR* clinical benefit rate, *MTD* maximum tolerated dose

^a^High-risk by IMWG criteria (del 17p, t(4;14), t(14;16), +1q21, −1p, hypodiploidy, and/or deletion 13q by conventional cytogenetics)

The median PFS for all patients (*N* = 64) treated in the study was 4.8 months (95% CI: 2.8–9.7 months, Fig. 1a). PFS in patients refractory to carfilzomib was 2.2 months vs. 8.4 months in carfilzomib non-refractory patients (Fig. 1b). In patients dual-refractory to a PI and IMiD, PFS was 2.8 months vs. 13.8 months in non-dual-refractory patients. PFS in patents with 1–2 lines of prior therapy was not reached, and 3.5 months in patients with ≥3 lines of prior therapy (Fig. 1c). In 14 patients treated at the MTD of filanesib and carfilzomib in the Part B dose-expansion cohort, median PFS was 4.7 months (95% CI: 2.5–8.4 months). The median OS for all patients treated on study was 24.9 months (95% CI: 17.5–47.1 months, Supplementary Fig. S2) at a median follow-up time of 49.7 months.

**Fig. 1:**
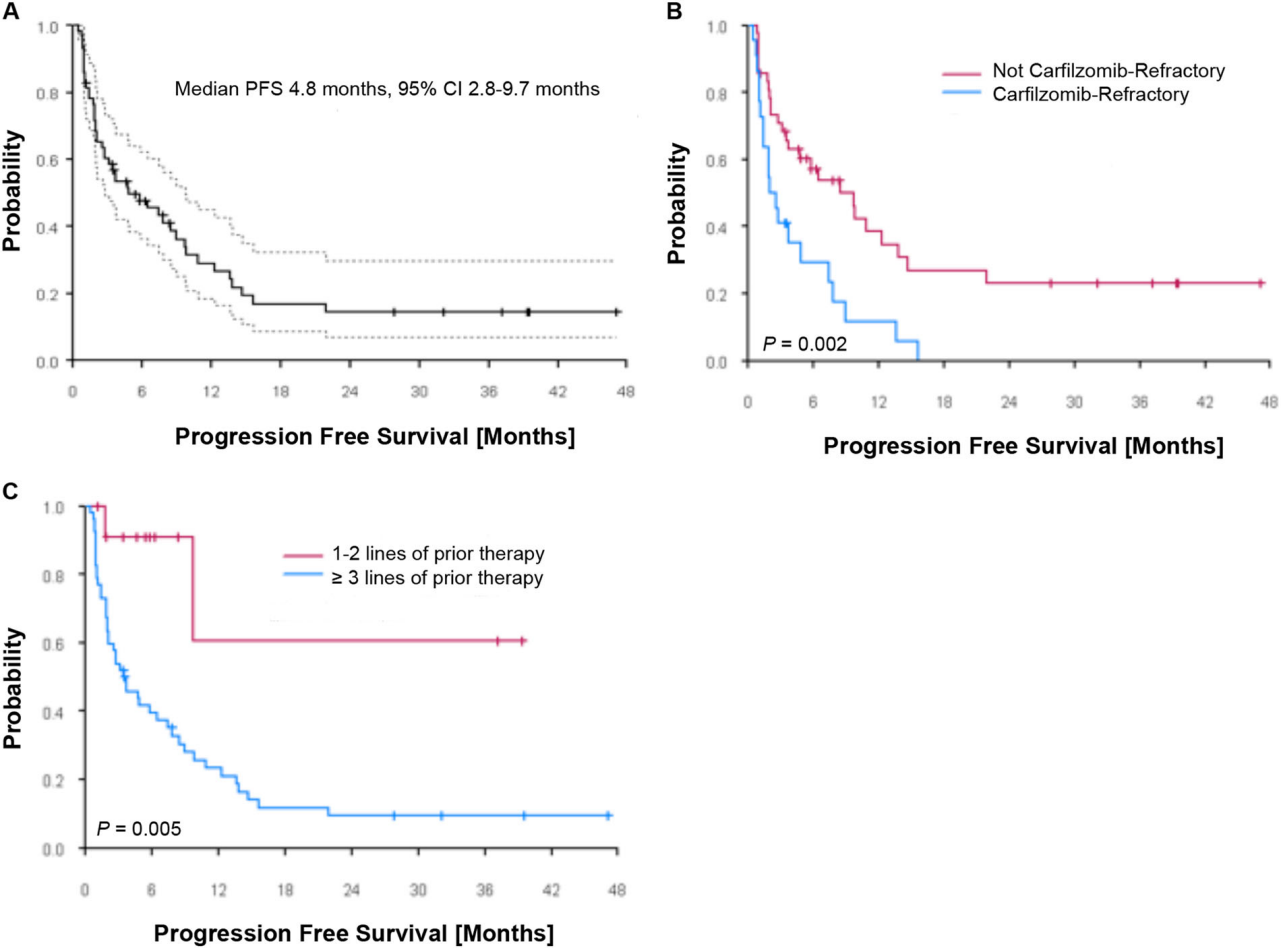
Progression-free survival (PFS) in **a**) all patients treated on study, **b**) patients stratified based on carfilzomib non-refractory or refractory status, and **c**) patients stratified by 1–2 lines or ≥3 lines of prior therapy

In summary, this phase 1 study with filanesib, carfilzomib, and dexamethasone demonstrates that filanesib and carfilzomib can be combined safely at the MTD of the individual drugs^4,7^ with an expected and manageable AE profile. However, efficacy was marginal in the context of today’s available therapeutic options for relapsed and/or refractory myeloma, and the measurable benefit of the addition of filanesib to carfilzomib and dexamethasone is uncertain without a randomized study. Further evaluation of predictor biomarkers such as low baseline serum alpha 1-acid glycoprotein (AAG) levels would be important to help identify myeloma patients most likely benefit from KSP inhibition with filanesib-based therapies^4,8^.

## Supplementary information

Supplemental Material
