# Supplementary material for: A phase 1 study of filanesib, carfilzomib, and dexamethasone in patients with relapsed and/or refractory multiple myeloma
Source: Blood Cancer J. 2019 Oct 1;9(10):80. doi: 10.1038/s41408-019-0240-6 (PMC6773683; doi:10.1038/s41408-019-0240-6)
Supplement: Supplementary file 1 — Supplemental Material [file 41408_2019_240_MOESM1_ESM.docx]

**SUPPLEMENTARY METHODS**

**Study Design**

This phase 1 open label, single-center study evaluated the safety and MTD of the combination of filanesib, carfilzomib, and dexamethasone in relapsed and/or refractory myeloma. The study sponsor was The University of Texas MD Anderson Cancer Center (UTMDACC) with study funding from Array BioPharma and Onyx Pharmaceuticals (Amgen, Inc.), who also provided filanesib and carfilzomib, respectively. All enrolled patients provided written informed consent, and the trial was conducted in accordance with the principles of the Declaration of Helsinki and the International Conference on Harmonization Good Clinical Practice guidelines. The study was registered at ClinicalTrials.gov (NCT01372540).

There were two parts to the study (Supplementary Figure S1). In Part A, filanesib was dose-escalated in a 3+3 design with a fixed-dose of carfilzomib and dexamethasone (Supplementary Table S1). Once the MTD was established for Part A, a two-cohort expansion phase was conducted with a targeted enrollment of 14 patients who were carfilzomib-sensitive and 14 patients who were carfilzomib-refractory.

In Part B, additional dose-escalation was then performed with carfilzomib in a 3+3 design with the MTD dose of filanesib established in Part A. However, if a particular carfilzomib dose level was not considered safe (≥2/6 or ≥2/3 DLTs) in combination with filanesib, then the filanesib dose would be deescalated to one dose level lower than MTD from Part A, while keeping carfilzomib at the same dose level (Supplementary Table S1). After establishment of the MTD in Part B, a dose-expansion cohort was then initiated with a planned enrollment of 28 additional patients at the Part B MTD. The data cut-off for the final analysis was November 5, 2018.

**Patients**

Patients with relapsed/and or refractory myeloma with at least one prior line of therapy, including at least one full cycle of a PI and an IMiD, were eligible for this study. Other inclusion criteria included measurable disease defined as a serum monoclonal protein ≥0.5 g/dL, urine M-protein ≥200 mg/24 hour, or involved serum free light chain (FLC) level ≥10 mg/dL. Patients with oligo-secretory or non-secretory myeloma were eligible if a bone marrow showed ≥ 30% plasmacytosis. Required hematologic parameters included an absolute neutrophil count ≥1.5 x 10^9^/L and platelets ≥75 x 10^9^/L unless the bone marrow plasmacytosis ≥50%, in which case a platelet count of ≥50 x 10^9^/L was allowed. Adequate renal function defined as a serum creatinine ≤2.5 mg/dl and hepatic function defined as a total bilirubin ≤2.0 mg/dL and AST (SGOT) and ALT (SGPT) <2.5x the upper limit of normal were also required.

**Safety and Efficacy Evaluation**

Safety assessments were performed prior to each cycle of therapy with physical exam and review of clinical laboratory tests and adverse events (AEs). Toxicity was assessed as per the National Cancer Institute Common Terminology Criteria for Adverse Events v4.0. During the Part A and Part B dose-escalation phases, patients were evaluated for dose-limiting toxicities (DLTs) during the DLT-evaluable period during cycle 1. Hematologic DLTs were defined as grade 4 neutropenia lasting >7 days, febrile neutropenia, grade 4 thrombocytopenia lasting >7 days and non-responsive to platelet transfusions (i.e., not improving to grade ≤ 3 thrombocytopenia), or any grade of thrombocytopenia associated with ≥Grade 3 bleeding attributed to filanesib and/or carfilzomib.

Non-hematological DLTs were defined as any grade 3 or 4 AE with the exception of nausea and/or vomiting in the absence of maximal support with anti-emetic prophylaxis, diarrhea in the absence of maximal support with anti-diarrheal agents, hyperglycemia, and clinically asymptomatic laboratory abnormalities.

Treatment efficacy was evaluated prior to each cycle, and overall response (ORR, ≥PR) and disease progression were assessed by International Myeloma Working Group (IMWG) Uniform Response Criteria. Patients who had at least one-post treatment disease assessment were considered evaluable for response.

**Statistical Analysis**

During the 3+3 dose-escalation phases of Part A and Part B of the study, the MTD for filanesib or carfilzomib were defined as the highest dose level where <2 DLTs were observed among 6 patients. For the dose-expansion phase of Part B, the regimen was considered to be promising if the ORR after 2 cycles was at least 30%, and the DLT rate after 1 cycle was below 30%. A sample size of 28 ensured that, if the trial was not terminated early, a posterior 90% credibility interval for ORR after 2 cycles would have width of .27 at most, under the assumption of an ORR of 30%. Patients were monitored in cohort sizes of 14 according to the following stopping boundaries for ORR after 2 cycles and DLTs after 1 cycle. Using predetermined stopping boundaries, the study would be stopped if ≤1 PR or better or ≥8 DLTs were observed after the first 14 patients were evaluated.

The Kaplan-Meier method was used to estimate time-to-event outcomes including progression-free survival (PFS) and overall survival (OS). PFS was defined as the time from initiation of therapy to disease progression or death from any cause, whichever occurred first. OS was defined from the date of treatment initiation to the date of death or to date of last follow-up. Patients who had not progressed at the time of last disease evaluation or prior to next therapy were censored for PFS. The log-rank test was used to evaluate the difference in time-to-event outcomes between patient groups. Statistical software SAS 9.3 (SAS, Cary, NC) and TIBCO Spotfire S+ 8.2 (TIBCO Software Inc., Palo Alto, CA) were used for all the analyses.

**SUPPLEMENTARY FIGURE S1.** Study design.

**
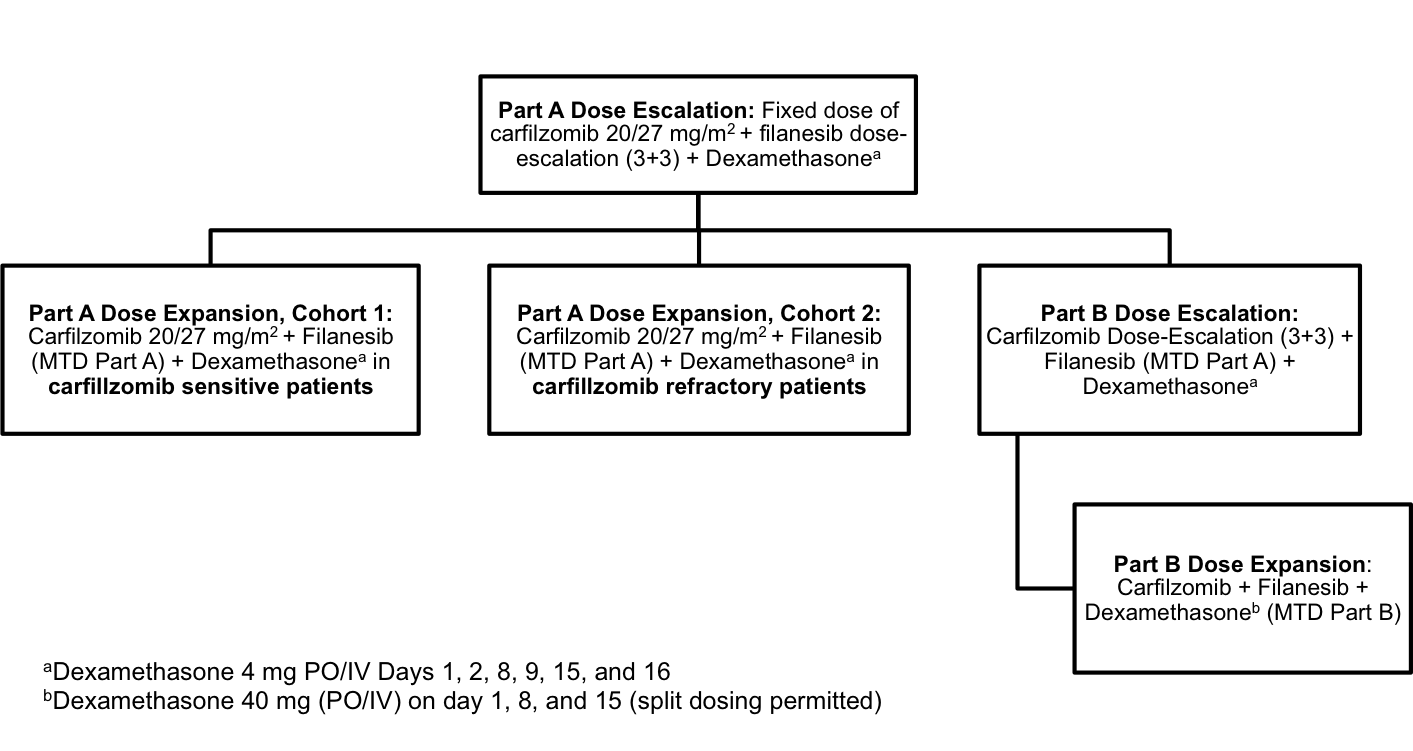
**

**SUPPLEMENTARY FIGURE S2.** Overall survival (OS**)** in all patients treated on study.


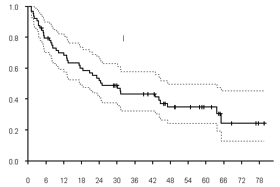


**Overall Survival [Months]**

**Probability**

Median OS 24.9 months, 95% CI 17.5-47.1 months

**SUPPLEMENTARY TABLE S1.**  Part A and Part B Treatment Dose Levels and Schedule (28-day cycle)

| **Part A** | **Filanesib mg/m2 IV Days 1, 2, 15, and 16** | **Carfilzomib* mg/m2 IV Days 1, 2, 8, 9, 15, and 16** | **Dexamethasone mg PO/IV Days 1, 2, 8, 9, 15, and 16** |
| --- | --- | --- | --- |
| **Dose Level -1** | 0.5 | 20/27 | 4 |
| **Dose Level 1** | 0.75 | 20/27 | 4 |
| **Dose Level 2** | 1 | 20/27 | 4 |
| **Dose Level 3** | 1.25 | 20/27 | 4 |
| **Dose Level 4** | 1.5 | 20/27 | 4 |
| **Part B** | **Filanesib mg/m2 IV Days 1, 2, 15, and 16** | **Carfilzomib* mg/m2 IV Days 1, 2, 8, 9, 15, and 16** | **Dexamethasone mg PO/IV Days 1, 2, 8, 9, 15, and 16** |
| **Dose Level 1a** | One dose level lower than MTD from Part A | 20/36 | 4 |
| **Dose Level 1** | MTD from Part A | 20/36 | 4 |
| **Dose Level 2a** | One dose level lower than MTD from Part A | 20/45 | 4 |
| **Dose Level 2** | MTD from Part A | 20/45 | 4 |
| **Dose Level 3a** | One dose level lower than MTD from Part A | 20/56 | 4 |
| **Dose Level 3** | MTD from Part A | 20/56 | 4 |
| *Carfilzomib dosed at 20 mg/m2 on Cycle 1, Day 1 and 2. All subsequent infusions at the higher dose (as assigned in each cohort). | | | |

**SUPPLEMENTARY TABLE S2.** Baseline Patient and Disease Characteristics

| **Median Age, (range)** | 63 | (41-84) |
| --- | --- | --- |
| **Sex** |  |  |
| **Male, N (%)** | 39 | (61) |
| **Female, N (%)** | 25 | (39) |
| **Median lines of therapy, (range)** | 5 | (1-13) |
| **1 line, N (%)** | 5 | (8) |
| **2 lines, N (%)** | 7 | (11) |
| **≥3 lines, N (%)** | 52 | (81) |
| **Previous Therapy** |  |  |
| **Prior Lenalidomide, N (%)** | 64 | (100) |
| **Prior Pomalidomide, N (%)** | 17 | (27) |
| **Prior Bortezomib, N (%)** | 62 | (97) |
| **Prior Carfilzomib, N (%)** | 23 | (36) |
| **Refractory to Lenalidomide, N (%)** | 48 | (75) |
| **Refractory to Pomalidomide, N (%)** | 10 | (16) |
| **Refractory to Bortezomib, N (%)** | 37 | (58) |
| **Refractory to Carfilzomib, N (%)** | 22 | (34) |
| **Refractory to IMiD and Proteasome Inhibitor, N (%)** | 40 | (63) |
| **Prior autologous stem cell transplantation** | 56 | (88) |
| **High risk Cg/FISH (R-ISS), N (%)*** | 6 | (13) |
| **High risk Cg/FISH (IMWG), N (%)°** | 17 | (35) |
| *Among 48 evaluable patients |  |  |
| °Among 49 evaluable patients |  |  |

**SUPPLEMENTARY TABLE S3.** Treatment Emergent Adverse Events at Part A MTD and Part B MTD

|  | **Part A MTD, N=30** | | | **Part B MTD, N=14** | | |
| --- | --- | --- | --- | --- | --- | --- |
| **Adverse Event (AE)*** | **All Grades** | **Grade 3** | **Grade 4** | **All Grades** | **Grade 3** | **Grade 4** |
| **Hematologic Events N (%)** | | | | | | |
| Anemia | 20 (67) | 10 (33) | 1 (3) | 11 (61) | 4 (22) |  |
| Neutrophil count decreased | 24 (80) | 11 (37) | 13 (43) | 11 (61) | 2 (11) | 4 (22) |
| Platelet count decreased | 26 (87) | 13 (43) | 5 (17) | 13 (72) | 9 (50) | 4 (22) |
| White blood cell decreased | 27 (90) | 13 (43) | 5 (17) | 13 (72) | 4 (22) | 3 (17) |
| **Non-Hematologic Events N (%)** | | | | | | |
| Acute kidney injury |  |  |  |  | 1 (6) |  |
| ALT increased | 7 (23) | 1 (3) |  | 3 (17) | 1 (6) |  |
| Alkaline phosphatase increased | 12 (40) |  |  | 5 (28) |  |  |
| AST increased | 6 (20) | 1 (3) |  | 5 (28) | 1 (6) |  |
| Bilirubin increased | 6 (20) | 1 (3) |  | 4 (22) |  |  |
| Blurred vision | 10 (33) |  |  | 4 (22) |  |  |
| Cardiac disorders |  | 1 (3) |  |  |  |  |
| Constipation | 10 (33) |  |  |  |  |  |
| Creatinine increased | 11 (37) |  |  | 5 (28) |  |  |
| Diarrhea | 18 (60) |  |  | 6 (33) |  |  |
| Dizziness | 14 (47) | 2 (7) |  | 3 (17) |  |  |
| Dry eye | 7 (23) |  |  |  |  |  |
| Dyspnea | 13 (43) | 3 (10) |  | 6 (33) | 2 (11) |  |
| Edema limbs | 6 (20) |  |  | 3 (17) |  |  |
| Fatigue | 22 (73) | 3 (10) |  | 8 (44) |  |  |
| Febrile neutropenia |  | 1 (3) |  |  |  |  |
| Fever | 8 (27) |  |  |  |  |  |
| Generalized muscle weakness |  |  |  |  | 1 (6) |  |
| Hearing impaired |  |  |  |  | 1 (6) |  |
| Hyperglycemia |  |  |  | 4 (22) |  |  |
| Hyperkalemia | 5 (17) |  |  | 6 (33) |  |  |
| Hyperuricemia | 6 (20) |  |  | 7 (39) |  |  |
| Hypoalbuminemia | 10 (33) |  |  | 5 (28) |  |  |
| Hypocalcemia |  |  |  | 3 (17) | 1 (6) |  |
| Hypokalemia | 5 (17) |  |  | 4 (22) |  |  |
| Hypomagnesemia | 8 (27) |  |  | 6 (33) |  |  |
| Hypophosphatemia |  | 1 (3) |  | 3 (17) | 1 (6) |  |
| Infections and infestations |  | 1 (3) |  |  |  |  |
| Lipase increased | 8 (27) | 4 (13) |  |  |  |  |
| Lung infection |  | 3 (10) |  |  | 1 (6) |  |
| Memory impairment | 10 (33) |  |  | 3 (17) |  |  |
| Mucositis oral | 10 (33) | 1 (3) |  | 3 (17) |  |  |
| Myalgia | 16 (53) | 2 (7) |  | 5 (28) |  |  |
| Nausea | 13 (43) |  |  | 5(28) | 1 (6) |  |
| Peripheral sensory neuropathy | 8 (27) |  |  |  |  |  |
| Serum amylase increased | 11 (37) | 1 (3) |  |  |  |  |
| Syncope |  | 1 (3) |  |  |  |  |
| Urinary tract infection |  | 1 (3) |  |  |  |  |
| Vomiting | 6 (20) |  |  | 5 (28) |  |  |
| *AEs of any grade occurring in at least 15% of patients and all grade 3 and 4 AEs | | | | | | |
